# From NAFLD to MASLD: updated naming and diagnosis criteria for fatty liver disease

**DOI:** 10.1016/j.jlr.2023.100485

**Published:** 2023-12-14

**Authors:** Mary E. Rinella, Silvia Sookoian

**Affiliations:** 1 Pritzker School of Medicine, University of Chicago, Chicago, IL, USA; 2 Clinical and Molecular Hepatology, Centro de Investigación Traslacional en Salud, Universidad Maimónides, Buenos Aires, Argentina; 3 Facultad de Ciencias de la Salud, Universidad Maimónides, Buenos Aires, Argentina; 4 Consejo Nacional de Investigaciones Científicas y Técnicas (CONICET), Buenos Aires, Argentina

Metabolic dysfunction-associated steatotic liver disease (MASLD), formerly known as nonalcoholic fatty liver disease (NAFLD), is a prevalent chronic liver disease worldwide, affecting more than 30% of the global population (1, 2). Importantly, MASLD is a multifaceted disorder resulting from complex interactions with various cardiometabolic and environmental risk factors (3, 4, 5).

Nonalcoholic steatohepatitis (NASH), now replaced with the term metabolic dysfunction-associated steatohepatitis (MASH), was initially coined in 1980 to denote a condition exhibiting the histological traits of alcohol-associated liver disease in individuals who neither consume alcohol nor have any other liver disease of clinical importance (6, 7). Since then, there has been a surge in interest around MASLD, primarily because of its rapidly escalating global prevalence and the consequent research into its pathophysiological, clinical, and socioeconomic implications. Today, with advances in our understanding of disease pathophysiology, it is clear that while there is a clear underpinning of insulin resistance and adipose tissue dysfunction, significant heterogeneity exists, characterized by varying rates of progression and varied response to treatment (8, 9). These outcomes can be influenced by physiological and environmental factors alongside genetic predisposition (10). Advances in disease pathogenesis provide insight into potentially viable treatment targets, currently under clinical development (11, 12) and reliable disease biomarkers (13). Nonetheless, despite these advances in our understanding of dominant driving factors, the name of the disease remained unchanged from when it was first introduced 40 years ago.

It was crucial for the global liver community to adopt a revised nomenclature that acknowledged the root cause of disease and provide diagnostic criteria, while using nonstigmatizing language. The Nomenclature Development Initiative's global members' main objective was to establish revised nomenclature that could be implemented worldwide, raise disease awareness, and direct research and funding to save more lives (14, 15, 16).

## What was the Impetus for the Name Change?

While the term “nonalcoholic” is commonly used, it has been acknowledged that it does not accurately reflect the current understanding of disease drivers. Enough knowledge exists about the pathophysiology of NAFLD to move from a diagnosis of exclusion to one defined by specific criteria. Furthermore, the terminology was considered stigmatizing to some and potentially disparaging to those who suffer with alcohol-associated/related liver disease. In addition, the terminology “fatty” to describe a medical condition can be stigmatizing for some patients. As part of the Delphi process undertaken to reconsider NAFLD nomenclature, 61% and 66% of respondents found the terms “nonalcoholic” and “fatty” potentially stigmatizing (14, 15, 16), respectively. While the extent of perceived stigma will vary across individuals and cultures and thus highly dependent on who is asked and how they are asked the question, it is clear that if the use of stigmatizing language can be avoided, it should, since no level of stigma should be deemed “acceptable” (14, 15, 16, 17).

Alcohol intake is common in many cultures, and this left many patients outside the NAFLD diagnostic category, which limits daily intake to <20 g/30 g for females and males, respectively. Such patients were explicitly excluded from therapeutic trials and biomarker consortia, despite being at higher risk for adverse liver-related outcomes and all-cause mortality (1, 18, 19). Furthermore, there is a growing recognition that there are overlapping biological processes that may contribute to both NAFLD and alcohol-related liver disease (ALD) (20).

**Fig. 1:**
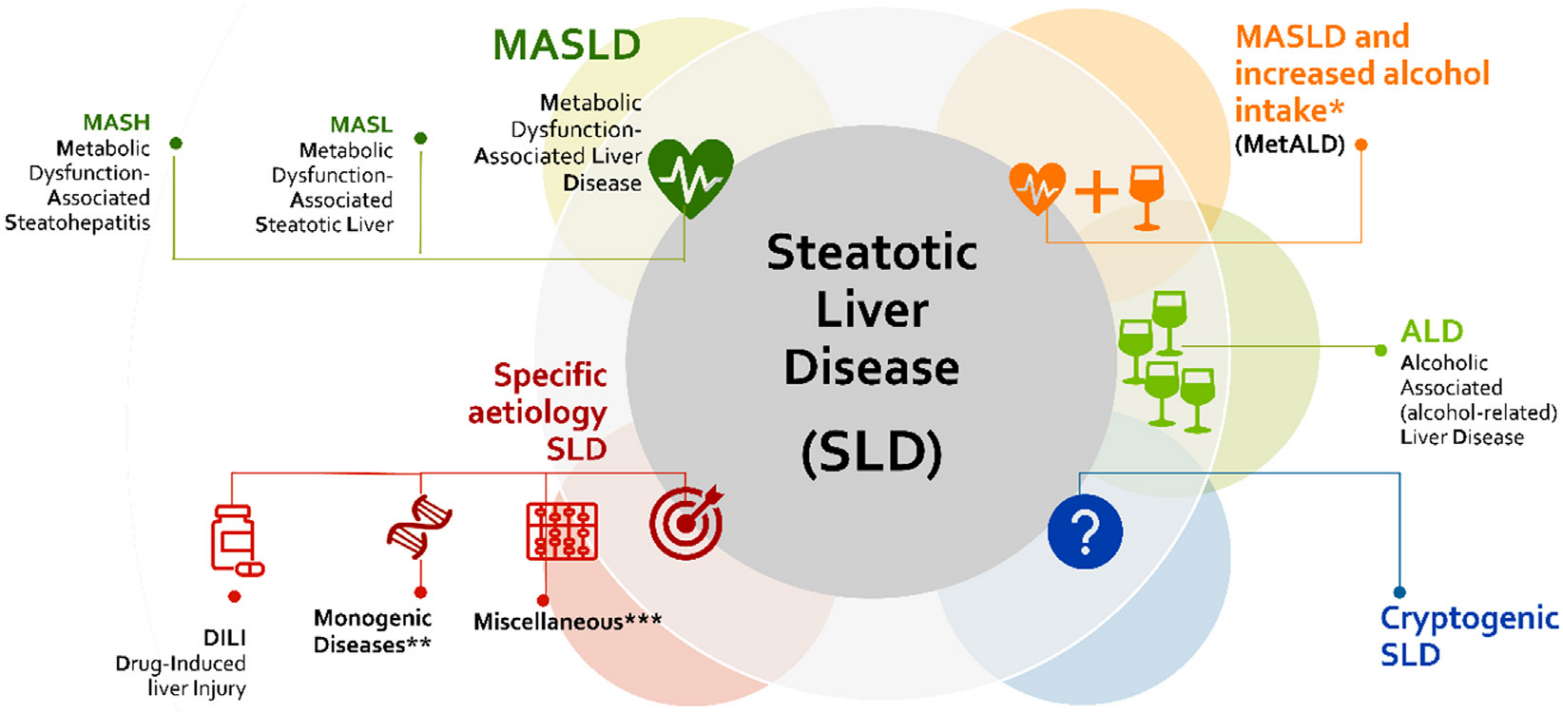
SLD was chosen as the overarching term to encompass the various causes of steatosis. NAFLD will now be called MASLD, which includes patients with hepatic steatosis and at least one of five cardiometabolic risk factors (adult and pediatric can be found elsewhere (14, 15, 16)). MASH replaces NASH in referring to metabolic dysfunction-associated steatohepatitis. The term steatohepatitis was considered an important pathophysiological concept that should be retained. A new category, MetALD, describes individuals with MASLD who consume more than 140 g/week of alcohol for women and 210 g/week for men. If additional factors contributing to steatosis are discovered, then this aligns with a combined etiology. When it comes to alcohol, the condition is referred to as MetALD or ALD, depending on the severity of alcohol consumption. If no overt cardiometabolic criteria are present, other causes should be ruled out. If none are identified, this is called cryptogenic SLD. Depending on clinical judgment, it may also be considered possible MASLD and would benefit from periodic reassessment on a case-by-case basis. ∗Weekly intake 140–350 g female, 210–420 g male. ∗∗e.g.: lysosomal acid lipase deficiency (LALD), Wilson disease, hypobetalipoproteinemia, and inborn errors of metabolism. ∗∗∗Hepatitis C virus, malnutrition, celiac disease, and human immunodeficiency virus.

NAFLD was an overarching term that encompassed numerous causes of steatosis, though what was managed as NAFLD/NASH represented a fairly specific disease entity characterized by the presence of cardiometabolic risk factors. Thus, the revised nomenclature selected an overarching term of steatotic liver disease (SLD) to parse out MASLD (what was considered true “NAFLD”) from other causes of steatosis, for example, hypobetalipoproteinemia, celiac disease, and so on, as well as mixed etiology conditions, such as overlap with alcohol, and alcohol-associated liver disease as well (Fig. 1).

## How was Consensus Achieved?

The desire to address the limitations of the existing nomenclature of NAFLD led to the development of a global collaborative multistakeholder effort under the leadership of the American Association for the Study of Liver Diseases and the European Association for the Study of the Liver in conjunction with the Asociación Latinoamericana para el Estudio del Hígado and other organizations (14, 15, 16). Experts from diverse fields, including hepatology, gastroenterology, pediatrics, endocrinology, hepatopathology, public health, and obesity, partnered with colleagues from industry, regulatory agencies, and patient advocacy organizations to develop questions to be addressed by the full member panel. The process was guided by a group deeply rooted in Delphi methodology (19, 21). They worked together to develop a consensus on revisions to the nomenclature and diagnostic criteria for this condition. The 33-member steering committee developed a four-part Delphi survey, in combination with two large in-person meetings to discuss points of controversy across a 3 year period. Members of the full Delphi panel represented 56 countries and spanned several relevant sectors, as previously noted. The committee identified five essential areas to consider when revising nomenclature: *1*) Can shortcomings of the current nomenclature be addressed? *2*) How important is steatohepatitis in disease definitions and endpoints? *3*) How should the role of alcohol be considered? *4*) How would renaming affect disease awareness, clinical trials, and regulatory approval processes? *5*) Can a new name decrease heterogeneity and facilitate future advancements? (14, 15, 16).

## What was the Output of the Consensus Process?

The Delphi panel defined a priori the consensus threshold of a ≥67% supermajority, except for two instances; considering the presence of stigma (for reasons noted above) and deciding whether to retain or change the current definition, once a supermajority had decided to revise the nomenclature, since it would have been illogical to redefine using affirmative language without providing criteria to diagnose it. The priorities in outlining the diagnostic criteria were to err on the side of being inclusive, to avoid large numbers of uncategorized patients, and thus to assure that decades of natural history studies in NAFLD or those in biomarker registries would refer to the same population (14, 15, 16). Since the publication of the nomenclature consensus document, comparative studies in various settings, including registries, population studies, community cohorts, and primary care settings, have noted near complete capture of the prior NAFLD population, using the definition for MASLD (22, 23, 24).

The consensus was to change the definition of NAFLD to MASLD, which now requires the presence of at least one of five cardiometabolic risk factors in the context of hepatic steatosis (Fig. 1). The caveat exists, as it did for NAFLD in the setting of advanced fibrosis, where steatosis may no longer be present (14, 15, 16). SLD was chosen as the overarching term to encompass the various causes of steatosis. The term cryptogenic SLD was reserved for those not meeting criteria for MASLD or a specific alternative etiology, acknowledging that such patients may be reclassified in the future as more data emerge. The new names to not alter the characterization of fibrotic severity or alter the definition of steatohepatitis, thus MASH can replace NASH. While not the intent of the nomenclature process, it is noted that in the future, staging may not be limited to histology, rather it will likely evolve to noninvasive staging of disease. Thus, the current consensus process adheres to previous case definitions for steatohepatitis and disease stages. The diagnosis of MASLD/MASH with advanced fibrosis or cirrhosis, even when steatosis is absent, will be based on previously agreed-upon criteria for NASH cirrhosis. This also applies to patients with MetALD and ALD (Figure) with significant fibrosis who may not have steatosis but still have SLD as part of the overall nomenclature, reflecting the injury mechanism.

## Implications and Implementation—Where are We Now?

The new nomenclature enhances the previous “nonalcoholic” label and accurately connects this liver disease to metabolic factors, previously referred to as “the hepatic manifestation of the metabolic syndrome.” This fundamental conceptual shift brings numerous practical implications. When discussing the disease with patients, it is advantageous to present a clear and concise explanation based on the underlying cardiometabolic irregularities that are associated with insulin resistance and the patient's other conditions. This approach is more straightforward than using a diagnosis of exclusion and makes it easier for patients to comprehend. In addition, it aids in communicating the primary therapeutic steps to be taken, as well as the risk factors for disease progression, both from a liver-oriented and holistic standpoint.

It has been difficult to identify a suitable replacement that satisfies the required criteria. Finding a term that fully encompasses the intricacy of the disease has proven to be a challenge. Yet, it was demonstrated that MASLD overlaps almost entirely with the NAFLD population (22, 23). The new structure should allow for incorporation of emergent phenotypes as the field progresses.

Despite decades of research and the promise of future therapeutics, awareness of NAFLD has remained low. This can be attributed to several factors, including low provider awareness of which patients are at risk of disease progression and identification of those with established advanced disease and a misperception that fatty liver is a benign entity. This then translates to patients understanding that it is not a major concern. “Rebranding” using terminology that links to disease underpinnings offers the opportunity to highlight impactful interventions. The likely emergence in the near-term of the first Food and Drug Administration-approved treatment for NASH/MASH has heightened efforts to identify patients at risk for progression who may be eligible for treatment, which can and will be leveraged to increase awareness of the new nomenclature by fostering collaborative efforts between society and industry. Dissemination of the new nomenclature within and outside hepatology is critical, as is adoption by journals and medical societies. Thus far, the new nomenclature has been endorsed by over 75 societies, only months after publication. Work is underway to begin to incorporate changes into medical school curricula and other areas of medical education. The biggest barrier to full implementation is the time and effort required to change billing codes, for which national, as well as international, efforts have begun.

## Implications for Authors Submitting to the Journal of Lipid Research

Based on the above considerations, we encourage authors of papers who are planning to submit to the *Journal of Lipid Research* to use the updated nomenclature and specifically where human subjects are involved. Authors of papers reporting findings in preclinical and cell-based models of steatosis/inflammatory responses are encouraged to use the term steatotic rather than “fatty” in their qualitative descriptions.

## Conflict of interest

M. E. R. provides scientific consulting for Boehringer Ingelheim, Cytodyn, Histoindex, Intercept, GSK, Madrigal, Novo Nordisk, and Takeda over the past 24 months. No speakers’ bureaus or stock ownership reported. S.S. declares no conflicts of interest with the contents of this article.
